# Distribution of acyclovir in central nervous system compartments: a porcine pharmacokinetic model

**DOI:** 10.1128/aac.01811-24

**Published:** 2025-07-09

**Authors:** T. Mariager, J. H. Terkelsen, J. M. Guldbæk, M. D. Nielsen, J. Vestergaard, M. Bue, K. Öbrink-Hansen, R. Nau, C. R. Bjarkam, E. C. M. de Lange, H. Nielsen, J. Bodilsen

**Affiliations:** 1 Department of Infectious Diseases, Aalborg University Hospital 669295, Aalborg, Denmark; 2 Department of Neurosurgery, Aalborg University Hospital 53141, Aalborg, Denmark; 3 The ESCMID Study Group for Infectious Diseases of the Brain (ESGIB), European Society of Clinical Microbiology and Infectious Diseases, Basel, Switzerland; 4 Department of Orthopedic Surgery, Aarhus University Hospital 11297, Aarhus, Denmark; 5 Aarhus Denmark Microdialysis Research Group (ADMIRE), Aarhus University Hospital 11297, Aarhus, Denmark; 6 Department of Internal Medicine, Gødstrup Hospital 72751, Herning, Denmark; 7 Institute of Neuropathology, University Medical Center 27177, Göttingen, Germany; 8 Department of Clinical Medicine, Aalborg University 572587, Aalborg, Denmark; 9 Division of Systems Pharmacology and Pharmacy, Leiden Academic Center for Drug Research, Leiden University 195335, Leiden, the Netherlands; IrsiCaixa Institut de Recerca de la Sida, Barcelona, Spain

**Keywords:** acyclovir, CNS PK/PD, pharmacokinetics, antiviral agents, encephalitis, herpes simplex virus

## Abstract

Herpes simplex virus (HSV) encephalitis is a severe infection with high mortality and neurological sequelae if untreated. Intravenous acyclovir (ACV) is the standard treatment, but its central nervous system (CNS) penetration is not fully understood. To evaluate the distribution of ACV in various CNS compartments in a porcine pharmacokinetic model, 12 female pigs were divided into two groups: group I receiving a single ACV dose (10 mg/kg) and group II receiving three doses over 24 h. Microdialysis sampled unbound ACV concentrations in cortical and subcortical extracellular fluid (ECF), ventricular cerebrospinal fluid (CSF), and cisternal CSF. The ACV target concentration was defined as peak concentration (*f*C_max_)  > inhibitory concentration 50% (IC_50_) for HSV-1 at 0.56 µg/mL. Pharmacokinetic parameters, including *f*C_max_, time above IC_50_ (T > IC_50_), and area under the curve (AUC), were analyzed. The target ACV concentration (*f*C_max_ > 0.56 µg/mL) was achieved in all ECF and CSF compartments during the second and third dosing intervals. The T > IC_50_ and AUC increased from the first to the third dose and were consistent across compartments. Intracerebral penetration ratios (*f*AUC_tissue_/*f*AUC_plasma_) during the third dose ranged from 0.18 to 0.32 within the CNS compartments. In conclusion, ACV administered intravenously at 10 mg/kg every eighth hour achieved therapeutic levels in porcine CNS compartments after the second dose, suggesting that current dosing regimens are effective in treating HSV encephalitis. However, the first dose may not reach therapeutic levels, suggesting that higher initial dosages or prolonged infusions should be considered. Further studies under inflammatory conditions are warranted to extrapolate these findings.

## INTRODUCTION

Viral encephalitis caused by herpes simplex virus type 1 or 2 (HSV-1/2), or varicella-zoster virus, is a devastating infection characterized by high mortality rates and frequent sequelae (1). Two randomized controlled trials of HSV encephalitis demonstrated a reduction in mortality from 50% to 19% in patients treated with intravenous (IV) acyclovir (ACV) (2, 3).

Current guidelines for managing HSV encephalitis recommend IV ACV administered as boluses at 10 mg/kg every 8 h for patients with dose adjustments in patients with renal impairment (4–6). Using IV doses of 5–15 mg/kg, the mean plasma half-life has been estimated to range between 1.5 and 3 h and plasma concentrations between 6.7 and 20.6 µg/mL during steady-state (7). In neonates, higher dosages of ACV (20 mg/kg every 8 h) are recommended due to studies suggesting reduced risks of death and relapse as well as an increased likelihood of normal development compared to regimens using 10 mg/kg (6, 8, 9).

Although ACV is generally well-tolerated in adult patients, nephropathy and neuropsychiatric symptoms are potential dose-related, reversible side effects (8, 10). HSV and other herpes viruses may also exhibit variable or reduced susceptibility to acyclovir (9, 11). This variability is influenced by differences in viral strains, antigenic properties, and the specific host cell lines used for susceptibility testing (12). Comparable variations in susceptibility are also observed in plaque reduction assays according to choice of viral inoculum, incubation conditions, and assay standardization (12, 13). The intricacies associated with elevated risks of unfavorable clinical outcomes, the possibility of dose-dependent adverse effects, and the sporadic resistance to ACV underscore the imperative for precision in dosing during the treatment of HSV infections, particularly those involving the central nervous system, such as encephalitis.

Previous clinical studies of ACV penetration within the central nervous system (CNS) have mostly relied on measurements of concentrations within the lumbar cerebrospinal fluid (CSF) collected during routine clinical care, thus only representing static estimates with no strict timing between drug administration and sampling (7, 14–18) and without consideration of the state of the blood brain-barrier (BBB) and the blood-CSF barrier (19). This introduces uncertainty regarding the translational value of CSF concentrations to other areas of the brain (20).

Microdialysis is a unique method for continuous *in vivo* monitoring of unbound drug concentrations in multiple extracellular fluids (ECF) and CSF compartments simultaneously (21, 22). ACV has previously been validated as an appropriate *in vivo* substrate for microdialysis sampling (23–26). Using microdialysis in an established pharmacokinetic (PK) porcine CNS model (27), the present study aimed to describe the distribution of ACV within brain ECF and CSF during multiple dosing intervals.

## MATERIALS AND METHODS

The present study was approved by the Danish Animal Experiments Inspectorate (2020-15-0201-00401). The experimental procedures were carried out at the laboratory facilities of the Department of Biomedicine, Aalborg University Hospital, Denmark, with chemical drug analyses performed at BioXpedia A/S, Aarhus, Denmark.

### Study overview

**Fig 1:**
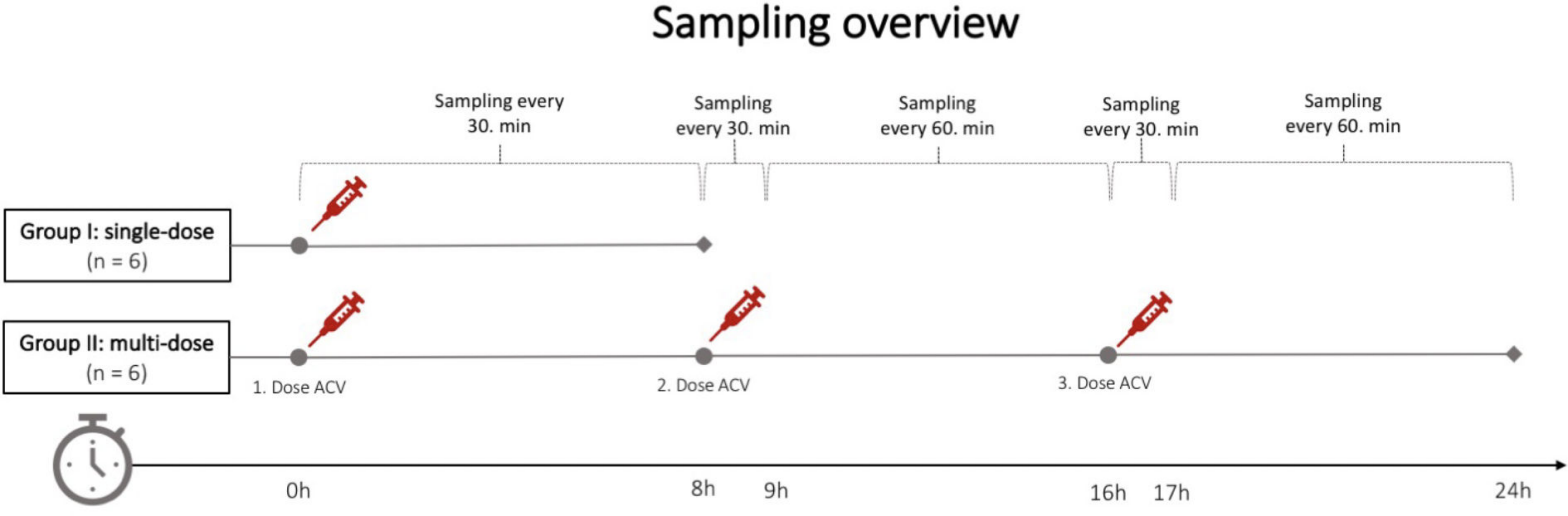
Microdialysis sampling overview of Groups I and II after intravenously administered acyclovir (10 mg/kg) in 12 pigs. Blood samples were collected at the midpoint of each microdialysis interval.

Twelve female pigs (Danish Landrace breed; weight range 37–48 kg, approximately 3 months of age) were included and equally distributed into two groups. Group I received a single bolus dose of weight-adjusted (10 mg/kg) ACV and was monitored for 8 h, while Group II received three doses of ACV with monitoring extended to 24 h (Fig. 1). The concentrations of unbound ACV were assessed within plasma, ECF, and CSF every 30th minute for the first 8 h in both groups. For Group II, sampling continued every 30th minute post-administration of the second and third doses until the 24th hour (Fig. 1).

### Definition of antiviral target

Plaque reduction assays are the most commonly used methods to determine the antiviral activity of different drugs. The drug concentration required to inhibit the number of infected cells by 50% is defined as the inhibitory concentration 50% (IC_50_) (11, 13, 28). The *in vitro* IC_50_ for ACV has previously been correlated with clinical efficacy for HSV infections (9). However, due to the significant heterogeneity of different HSV-1 strains and plaque assays, the reported IC_50_ ranges between 0.02 and 1.8 µg/mL (11, 28–30).

While recent literature suggests that maintaining drug concentrations above the IC_50_ for at least 50% of the dosing interval (T > IC_50_) may be the optimal target for antiviral efficacy (30), the majority of prior studies have used C_max_ as the primary pharmacodynamic target (11, 29, 30). Additionally, variability in viral susceptibility can arise from mutations in thymidine kinase and DNA polymerase genes, which can confer partial or complete ACV resistance, further complicating the establishment of a universal target concentration. To enhance comparability with these studies, we defined a target ACV concentration as *f*C_max_ >0.56 µg/mL. Additionally, T > IC_50_ data are presented in Table 1 to provide further insight into the time-dependent aspect of ACV efficacy.

**TABLE 1:** Key pharmacokinetic parameters for free unbound acyclovir in plasma, cortical brain ECF, subcortical forebrain ECF, ventricular CSF, cisterna magna CSF, and lumbar CSF^*a*^

	First dosing interval			Second dosing interval			Third dosing interval		
Parameter	N	Median	Range	N	Median	Range	N	Median	Range
*f*C_max_ (μg/mL)									
Plasma (unbound)	6	23.31	18.9–27.8	6	20.5	15.2–43.5	6	17.44	10.7–24.8
Ventricular CSF	11	0.24	0.07–2.18	5	1.05	0.41–1.23	5	0.80	0.30–1.15
Cisterna magna CSF	12	0.37	0.06–1.74	6	0.54	0.27–2.69	6	0.65	0.30–3.75
Lumbar CSF	11	0.67	0.03–1.66	5	0.73	0.61–5.71	5	0.96	0.45–6.59
Cortical ECF	12	0.28	0.02–1.65	6	0.90	0.30–1.73	6	0.93	0.25–1.38
Subcortical ECF	11	0.24	0.02–1.05	5	0.79	0.15–2.09	5	0.77	0.29–1.83
*f*T(h) > IC_50_									
Plasma (unbound)	6	1	0.5–1.0	6	1.2	0.50–1.3	6	1.0	0.5–1.3
Ventricular CSF	11	0	0–2.3	5	1.2	0–2.9	5	1.1	0–1.3
Cisterna magna CSF	12	0	0–3.9	6	0.7	0–4.1	6	1.1	0–3.8
Lumbar CSF	11	0.5	0–1.7	5	0.6	0.2–5.6	5	1.4	0–4.7
Cortical ECF	12	0	0–1.9	6	1.5	0–3.7	6	2.3	0–3.2
Subcortical ECF	11	0	0–1.2	5	0.9	0–4.3	5	1.2	0–4.5
*f*AUC (h × μg/mL)		*f*AUC_0-8_			*f*AUC_8-16_			*f*AUC_16-24_	
Plasma (unbound)	6	13.18	5.59–23.17	6	11.75	4.74–19.77	6	10.09	5.22–12.14
Ventricular CSF	11	0.63	0.20–3.00	5	1.23	0.64–3.77	5	1.39	0.43–3.24
Cisterna magna CSF	12	1.02	0.09–4.58	6	2.07	0.47–7.77	6	2.32	0.50–7.59
Lumbar CSF	11	1.27	0.05–2.03	5	1.59	0.50–13.28	5	2.26	0.43–12.8
Cortical ECF	12	0.68	0.07–2.31	6	2.00	0.64–4.51	6	2.77	0.75–4.16
Subcortical ECF	11	0.31	0.11–3.00	5	2.29	0.77–5.99	5	2.32	1.38–6.18
*f*T½ (h)									
Plasma (unbound)	6	0.5	0.3–2.3	6	0.4	0.2–1.0	6	0.4	0.4–0.6
Ventricular CSF	8	9.9	1.1–72	5	4.8	1.1–11.1	5	7.5	1.9–12.5
Cisterna magna CSF	10	10.9	4.0–47.7	6	8.4	1.9–20.0	6	8.2	1.4–22.1
Lumbar CSF	10	9.3	5.0–38.3	5	4.2	0.7–9.6	5	4.1	0.5–6
Cortical ECF	8	8.1	1.7–47.5	6	5.1	3.7–12.2	6	5.67	4.6–18.8
Subcortical ECF	9	20.8	4.2–62	5	6.7	2.5–19.1	5	7.88	3.3–28.9
*f*TOMC (h)									
Plasma (unbound)	6	0.3	0.3–0.3	6	0.3	0.3–0.3	6	0.3	0.3–0.3
Ventricular CSF	11	1.3	0.3–3.3	5	0.8	0.3–2.5	5	0.5	0.3–2.5
Cisterna magna CSF	12	1.3	0.3–2.3	6	0.8	0.3–1.5	6	0.8	0.3–1.5
Lumbar CSF	11	1.3	0.3–2.8	5	0.5	0.3–0.8	5	0.3	0.3–0.8
Cortical ECF	12	0.8	0.3–5.3	6	0.3	0.3–0.8	6	0.8	0.3–0.8
Subcortical ECF	11	0.8	0.3–3.8	5	0.5	0.3–1.5	5	0.8	0.3–1.5

^*a*^ *f*C_max_, peak drug concentration; *f*Time (h) > IC_50_, hours above 0.56 mg/mL; *f*AUC_0-8_, area under the concentration-time curve from 0–8 h; *f*AUC_8-16_, area under the concentration-time curve from 8–16 h; *f*AUC_16-24_, area under the concentration-time curve from 16–24 h; *f*T½, half-life (h); *f*TOMC, time (h) to *f*C_max_. *f*AUC, *f*C_max_, *f*T½, and *f*TOMC are given as medians. Acyclovir was administered intravenously every eighth hour for 24 h (10 mg/kg).

### Anesthesia and monitoring

The animals were anesthetized via intramuscular administration of Zoletil Vet (2 mL/10 kg), followed by endotracheal intubation and mechanical ventilation with a 0.2% sevoflurane mixture (Baxter, Illinois, USA). It should be noted that only female pigs were used in this study to minimize the physiological variation associated with the hierarchical behavior observed in male pigs. Subsequently, a 6 Fr sheath was inserted into the jugular vein for the following purposes: (i) continuous infusion of propofol (4 mg/kg/h), fentanyl (5 µg/kg/h), NaCl (0.9%) (10 mL/kg/h), and in Group II, continuous isotonic glucose (50 mg/mL); and (ii) administration of ACV. Following the initiation of intravenous sedation, sevoflurane administration was discontinued. Infusion rates were adjusted to maintain an appropriate level of sedation.

A 6 Fr sheath was percutaneously inserted into each femoral artery to enable continuous blood pressure monitoring and blood sampling. Body temperature was maintained within the normal range using forced-air warming blankets and was monitored via a bladder catheter equipped with a thermosensor. Arterial pH, blood glucose, and lactate levels were continuously monitored throughout the study. Blood pH was measured hourly and ranged between 7.43 and 7.63. Blood glucose levels were kept above 4 mmol/L using isotonic glucose (50 mg/mL), while blood lactate levels were maintained below 1.5 mmol/L by ensuring adequate oxygenation and ventilation. Vital parameters were recorded hourly.

### Sampling procedure

Microdialysis catheters were inserted into the cortical ECF, subcortical ECF, and ventricular CSF through an anterior craniotomy as recently described (31). Additionally, microdialysis catheters were inserted into the cisterna magna and lumbar cistern through the atlantooccipital membrane and the lumbar dura mater (32). Each CSF access was validated by aspirating CSF prior to the insertion of the catheter. A 30 min “no touch” period was completed after the placement of all catheters to allow for tissue equilibration. This 30 min interval was selected to balance practical constraints associated with completing the setup within the experimental timeframe. Next, all animals received a weight-adjusted IV bolus of ACV (Acyclovir, Pfizer, New York, USA) at a dose of 10 mg/kg, administered at time zero (dose range: 370 to 460 mg). ACV was administered manually as a bolus over 15 s to avoid potential variability in infusion rates that could arise with a standard 1 h infusion using a larger saline volume. Dialysates from each compartment were collected at 30 min intervals over 8 h in both Groups I and II, resulting in a total of 16 samples per animal after the first dose. In Group II, each animal received additional second and third doses of ACV, in which sampling was conducted after 30 min, after 60 min, and then every hour (Fig. 1).

Blood samples were collected at the midpoint of each microdialysis interval. Each blood sample was immediately cooled to 5°C in an ice bath for 15 min before centrifugation at 1,500 × *g* for 15 min. The resulting plasma aliquots and dialysates were manually transferred to clean tubes and placed directly on dry ice. Samples were transferred to a −80°C freezer until analysis within 3 h of study completion.

### Microdialysis

Microdialysis involves inserting a catheter with a semipermeable membrane into the targeted tissue, allowing extracellular analytes and water-soluble molecules to diffuse (21, 33). A continuously perfused solution collects these analytes, termed dialysate, which reflects a fraction of the analyte concentration in the tissue’s extracellular fluid. The absolute unbound analyte concentration can be quantified through calibration of the relative recovery using the “retrodialysis-by-drug” method (34), as described previously (27). Each concentration derived from microdialysis was attributed to the midpoint of its respective sampling interval. Microdialysis equipment was purchased from M Dialysis AB (Stockholm, Sweden), including 70 MD Brain microdialysis catheters (PA membrane, membrane length 10 mm, molecular cutoff 20 kDa). Each catheter was perfused using a precision pump (CMA 107, M Dialysis AB, Stockholm, Sweden) with 0.9% NaCl (Fresenius Kabi AB, Uppsala, Sweden) at a flow rate of 1 µL/min.

### Quantification of ACV

Free unbound ACV concentrations were determined in plasma and dialysates using high-pressure liquid chromatography-mass spectrometry with a lower limit of quantification (LLOQ) of 0.01 µg/mL in both plasma and dialysates. Protein precipitation and filtration were employed using the filter plate method (Strata-X 96 Method Development Well-Plate, 30 mg of X, X-C, X-CW, and X-AW, Ea) to quantify only the unbound fraction of ACV in plasma samples. Post hoc analysis revealed that total ACV concentrations had been measured in the plasma samples from Group I. Since it was impossible to determine the porcine protein binding reliably, the plasma samples from Group I were excluded. A complete description of the chemical analysis can be found in the Supplemental material.

### Pharmacokinetic analyses

All data were analyzed using Stata (v. 17, StataCorp LLC, Texas, USA). Peak drug concentration (*f*C_max_), time to *f*C_max_ (*f*TOMC), and half-life (*f*T_1/2_) were determined separately for each animal in all five compartments for each dosing interval. *f*C_max_ was defined as the highest observed concentration, *f*T_½_ was determined by log-linear regression, and the time (h) > IC_50_ was calculated using linear interpolation in Stata. All pharmacokinetic parameters are presented as medians with full range. Pairwise comparisons of these PK parameters were performed using the Wilcoxon signed-rank test, a non-parametric rank-based method that assesses the overall distribution of values. The area under the curve (*f*AUC) was calculated using the trapezoidal rule in each compartment for each dosing interval: *f*AUC_0-8_, *f*AUC_8-16_, and *f*AUC_16-24_. In cases where data were missing, linear interpolation was used to estimate the expected drug concentrations. As a measure of tissue penetration, the ratio of tissue *f*AUC_16-24_ to plasma *f*AUC_16-24_ was determined for Group II. We defined incomplete tissue penetration as an *f*AUC_tissue_/*f*AUC_plasma_ ratio <1.

## RESULTS

**Fig 2:**
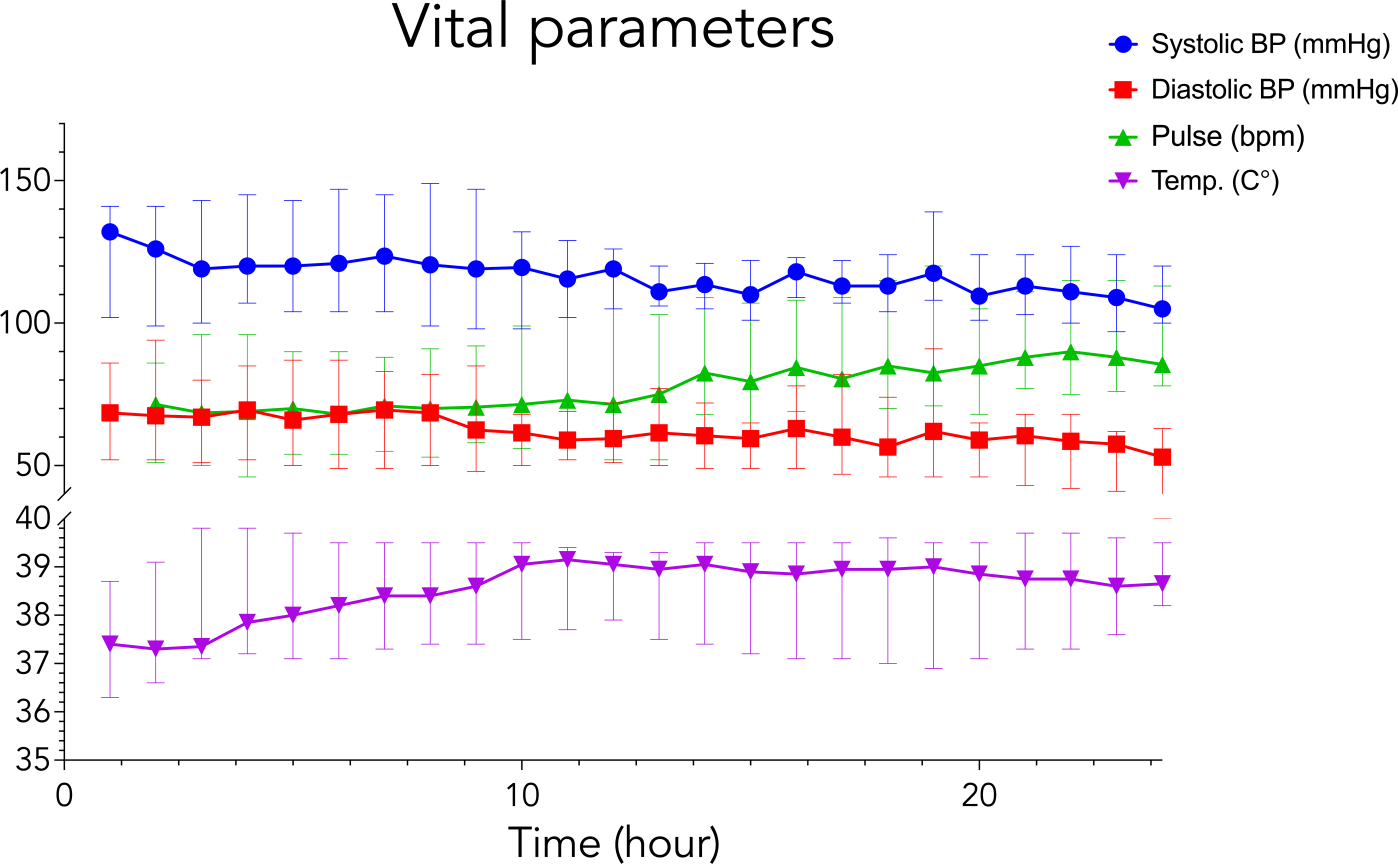
Mean vital parameters for animals in Group I and Group II. Bars represent the full range.

All animals completed the study period with stable vital parameters (Fig. 2). From 0 h to 24 h, the median temperature increased from 37.4°C to 38.7°C (*P* > 0.05) but did not exceed the upper limit of normal porcine temperature at 39.8°C (35). Both median systolic and diastolic blood pressures decreased during the experiment but remained within normotensive limits (35).

### Pharmacokinetic parameters

**Fig 3:**
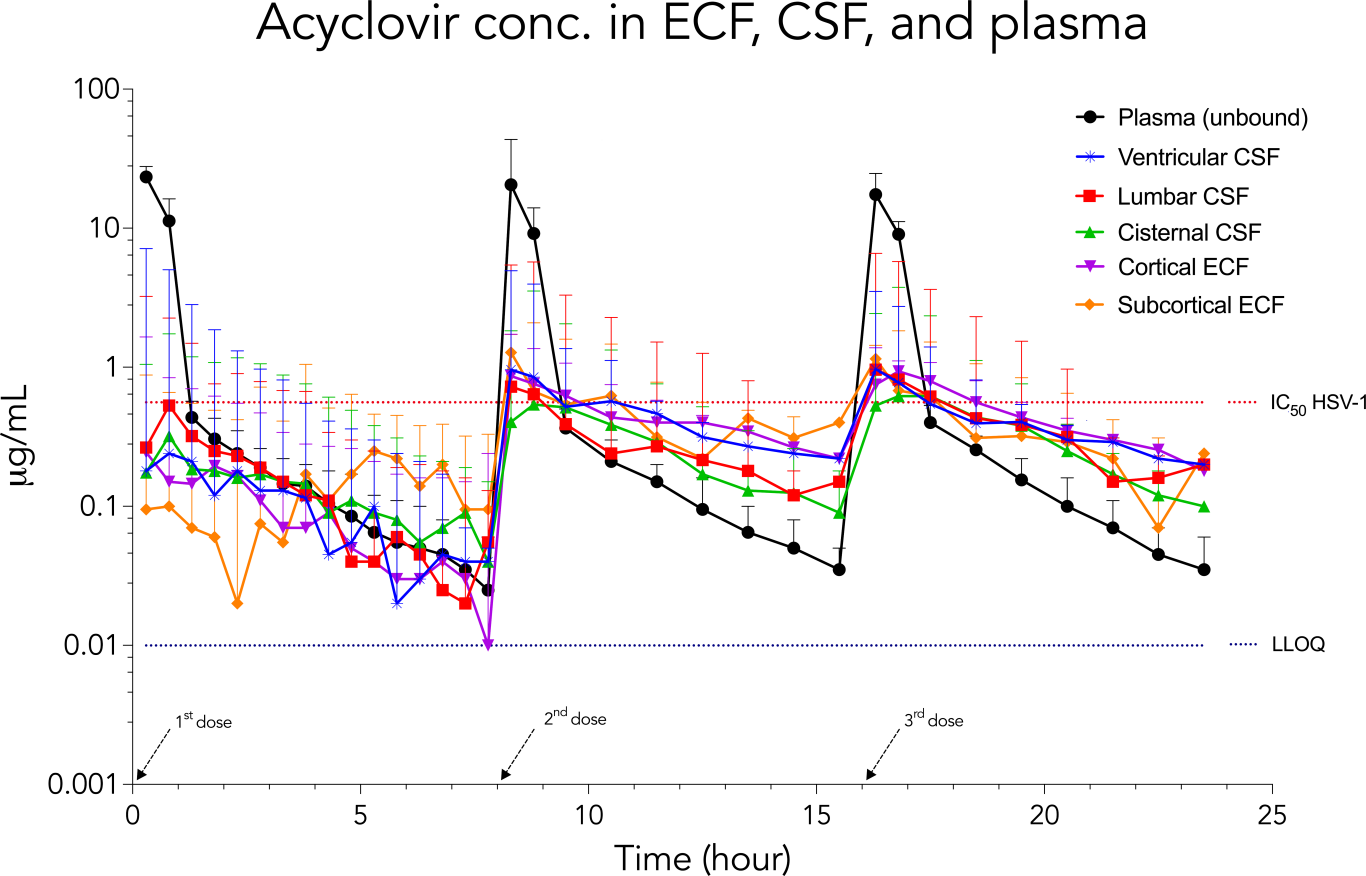
Median time-concentration profiles of unbound acyclovir within plasma, ventricular CSF, lumbar CSF, cisterna magna CSF, cortical ECF, and subcortical ECF. LLOQ, lower limit of quantification = 0.01 µg/mL. IC_50_, inhibitory concentration of 50% for HSV-1 = 0.56 µg/mL. Bars represent the full range.

Key pharmacokinetic parameters for each dosing interval are reported in Tables 1 and 2 and depicted in the concentration-time curve in Fig. 3.

**TABLE 2:** Tissue penetration ratios for free unbound acyclovir in various CSF and ECF compartments during the third dosing interval of acyclovir (10 mg/kg)^*a*^

	N	Median	Range
*f*AUC_tissue_/*f*AUC_plasma_ (third dose)			
Ventricular CSF	5	0.20	0.04–0.28
Cisterna magna CSF	6	0.27	0.05–0.66
Lumbar CSF	5	0.19	0.06–1.12
Cortical ECF	6	0.34	0.06–0.49
Subcortical ECF	5	0.32	0.11–0.54

^*a*^ *f*AUC_tissue_/*f*AUC_plasma_ = the tissue to plasma *f*AUC_16-24_ ratio during the third dosing of acyclovir. Acyclovir was administered intravenously every eighth hour for 24 h (10 mg/kg).

Median fC_max_ >0.56 µg/mL was achieved in all compartments during the second and third dosing intervals. In the cortical ECF, 4 out of 12 animals achieved the target fC_max_ >0.56 μg/mL during the first dosing interval, increasing to 5 of 6 animals by the third dosing interval. A similar trend was observed in the subcortical ECF, where 3 out of 11 animals met the target concentration during the first dosing interval, compared to 4 out of 5 animals in the third dosing interval.

During the first dosing interval, the median *f*C_max_ target was met in lumbar CSF and plasma (Table 1). Plasma consistently exhibited the highest median *f*C_max_ across all three dosing intervals. The median *f*C_max_ was similar between cortical ECF and subcortical ECF throughout all dosing intervals. During the third dosing interval, lumbar CSF median *f*C_max_ was significantly higher compared to both ventricular and cisterna magna CSF (*P* = 0.01 for both), though it remained similar to *f*C_max_ in cortical ECF (*P* = 0.13) and subcortical ECF (*P* = 0.06). A statistically significant increase in median *f*C_max_ from the first to the third dosing interval was observed in subcortical ECF (0.24 µg/mL vs 0.77 µg/mL; *P* = 0.04), cisterna magna CSF (0.37 µg/mL vs 0.65 µg/mL; *P* = 0.03), and lumbar CSF (0.67 µg/mL vs 0.96 µg/mL; *P* = 0.04). In contrast, increases in *f*C_max_ between the first and third dosing intervals were not significant for cortical ECF (0.28 µg/mL vs 0.93 µg/mL; *P* = 0.41) or ventricular CSF (0.24 µg/mL vs 0.80 µg/mL; *P* = 0.23).

The median *f*T > IC_50_ (0.56 µg/mL) demonstrated a trend to increase by the third dosing interval across all ECF and CSF compartments, though these changes compared to the first and second dose were not statistically significant. No significant intercompartmental differences were observed across all dosing intervals (Table 1). During the third dosing interval, the lowest median *f*T > IC_50_ was observed in plasma (1 h), whereas the highest median *f*T > IC_50_ was observed within cortical ECF (2.3 h). Within the CSF compartments, median *f*T > IC_50_ ranged between 1.1 and 1.4 h in the third dosing interval.

The highest *f*AUC was consistently observed in plasma across all dosing intervals. In the third dosing interval, the median *f*AUC_16–24_ was significantly higher in cortical ECF compared to ventricular CSF (2.77 h × μg/mL vs 1.39 h × μg/mL, respectively; *P* = 0.03). No other intercompartmental differences in *f*AUC were observed. A significant increase in median *f*AUC from the first to the third dosing interval was identified in cortical ECF (0.68 h × μg/mL vs 1.38 h × μg/mL; *P* = 0.03), cisterna magna CSF (1.02 h × μg/mL vs 2.32 h × μg/mL; *P* = 0.03), and lumbar CSF (1.27 h × μg/mL vs 2.26 h × μg/mL; *P* = 0.04). The median penetration ratio (*f*AUC_tissue_/*f*AUC_plasma_) during the third dosing interval ranged between 0.19 and 0.34 (Table 2).

The *f*T½ was significantly shorter in plasma compared to all ECF and CSF compartments across all dosing intervals (0.4–0.5 h vs 4.1–20.8 h, *P* = 0.03, *P* = 0.02, respectively) (Table 1). A significant reduction in *f*T½ was observed in lumbar CSF (9.28 h vs 4.10 h; *P* = 0.04) and in cisterna magna CSF (10.9 h vs 8.2 h; *P* = 0.04) from the first to the third dosing interval. Within ventricular CSF and both ECF compartments, no significant differences in *f*T½ were detected across the dosing intervals. During the third dosing interval, *f*T½ within lumbar CSF was significantly shorter than those observed within other CSF compartments (4.1 h vs 7.5–8.2 h; *P* = 0.00–0.01) and ECF compartments (4.1 h vs 5.7–7.9 h; *P* = 0.02–0.04).

The relative recovery (standard deviation) from the microdialysis was 53% (32) for ventricular CSF, 60% (32) for lumbar CSF, 72% (19) for cisterna magna CSF, 52% (31) for cortical ECF, and 57% (28) for subcortical ECF. In Group II, three microdialysis catheters malfunctioned and were excluded from analysis: one from the ventricular CSF, one from the lumbar CSF, and one from the subcortical ECF.

## DISCUSSION

To our knowledge, this is the first study to describe the *in vivo* distribution of ACV into different CNS compartments and across multiple doses. Our predefined target of a median *f*C_max_ > IC_50_ (0.56 µg/mL) was achieved in all ECF and CSF compartments during the second and third dosing intervals, but only in plasma and lumbar CSF during the first dosing interval. The corresponding *f*T > IC_50_ in ECF and CSF tended to increase from the first dose (range 0.5–1 h) to the third dose (1–2.3 h) and did not differ significantly across the compartments. These findings indicate that the 10 mg/kg dose regime efficiently achieves adequate ACV concentration within the brain ECF during the second and third dosing intervals for treating HSV-1 encephalitis. However, our data suggest that therapeutic levels for encephalitis may not be reached during the first dosing interval.

Although the *in vitro* IC_50_ has been correlated with the clinical response to antiviral treatment with ACV (9), this threshold has inherent limitations. Similar to bacterial minimum inhibitory concentrations, the IC_50_ can exhibit significant variability across different viral strains (28) and even within the same HSV lesion in the same patient (9). Therefore, using a single threshold to distinguish between appropriate and inappropriate ACV concentrations is somewhat arbitrary and may not necessarily serve as a universally applicable threshold to all patients. The inhibitory concentration of 90% (IC_90_) could offer a more conservative threshold; however, it has been demonstrated that achieving the IC_90_ can require ACV concentrations that are 10- to 100-fold higher than those needed for the IC_50_ (36). Furthermore, although there is no established consensus on the optimal PK/PD target for ACV and HSV-1, previous studies have suggested that the duration of time above the T > IC_50_ is correlated with better therapeutic efficacy (30). This is supported by ACV’s inhibitory mechanism against the viral DNA-polymerase at the cellular level (28). It has previously been reported that maximum efficacy is achieved when ACV concentrations exceed the IC_50_ for 50% of the dosing interval in plasma (37, 38). Given that the current *f*T > IC_50_ within ECF during the third dose corresponds to ACV levels above the IC_50_ for approximately 12%–28% of the dosing interval, an increased dose, the use of a loading dose, prolonged infusion, or continuous infusion may be considered to improve antiviral efficacy during the initial 24 h.

While no clinical trials or pharmacokinetic studies have directly compared continuous versus bolus ACV administration in this context, our data suggest that the administration of an increased loading dose may be beneficial. For example, based on our current uninfected model, an initial dose of approximately 20 mg/kg could potentially achieve therapeutic CNS concentration (i.e., C_max_ > IC_50_) already after the first dose and prolong the duration that ACV levels remain above the IC_50_. This approach is supported by the clinical practice for varicella zoster encephalitis, where a higher dose (15 mg/kg) is already recommended per IDSA guidelines (6), although this is associated with an increased risk of adverse events. In addition, continuous infusion of ACV has previously been used for HSV, Epstein-Barr virus, or cytomegalovirus infections in immunocompromised patients who did not respond to intermittent bolus ACV (38). In a hypothetical infected porcine PK model, where the BBB permeability is increased due to inflammation, the required loading dose might be lower owing to enhanced CNS penetration. These considerations underscore the need for further clinical trials and detailed pharmacokinetic studies to evaluate alternative dosing regimens, including the use of a loading dose and continuous infusion strategies, in order to optimize both C_max_ > IC_50_ and T > IC_50_ targets in the treatment of HSV encephalitis (39, 40).

Direct comparison of our ECF C_max_ to previous findings is limited to one study that found a *f*C_max_ of 10.8 µg/mL following IV ACV monitored for 180 min (25 mg/kg) in the rat brain (24). In this study, ACV penetration ratios within the brain ECF ranged from 0.32 to 0.34, which aligns with previous findings in rodent and canine brain ECF (24, 39, 40). Two of these studies analyzed ACV through microdialysis in rats or mice, but the observation period was limited to the initial 180 min of the first IV ACV dose. A previous clinical study of intracerebral ACV penetration reported brain-to-plasma ratios ranging from 0.25 to 0.70 (41). However, since these analyses were conducted on static homogenized brain tissue samples obtained post-mortem from three immunocompromised patients who died of cytomegalovirus pneumonia, direct comparison to our findings is not straightforward. Notably, our CSF penetration ratios (0.18–0.22) were comparable to those observed during steady-state conditions in patients with multiple sclerosis and intact BBB, following prolonged oral treatment with ACV or valacyclovir (14, 15).

Previous clinical studies of ACV have utilized lumbar CSF sampling via lumbar puncture as a surrogate for intracerebral concentrations (7, 15, 16). In the present study—the first to analyze the effects of multiple dosing intervals on ACV PK properties within ECF—we observed that lumbar CSF tended to overestimate ECF concentrations. These findings support prior recommendations to exercise caution when using lumbar CSF as a generalized proxy for drug concentrations within brain ECF (20, 42). However, during the third dosing interval, we found that the *f*C_max_ and *f*AUC in lumbar CSF were comparable to those in cortical ECF.

Additionally, a significant accumulation of ACV was observed in cortical and subcortical ECF AUC from the first to the third dosing interval. Several efflux pumps, including P-glycoprotein and organic anion transporter 3, have well-established affinities for ACV and affect its intracerebral penetration (24). The efficacy of these efflux pumps can be altered through chemical saturation, inflammatory processes, or circadian rhythmicity over time (43), which could affect ACV clearance from ECF in the current setup and explain the accumulation of ACV. Finally, diminished activity of intracellular metabolic enzymes, coupled with circadian variations in physiological processes within the CNS, may also contribute to the observed accumulation of ACV.

Notably, we sampled ACV in the frontal lobe rather than the temporal lobe, which is most often affected during HSV-1 encephalitis, due to practical constraints in accessing the porcine temporal region. Thus, any potential lobe-specific variation should be taken into account when interpreting the results.

### Limitations

This study presents several limitations. First, our data reflect a non-infected and non-inflamed BBB, which contrasts with the increased permeability observed during the acute phase of HSV-1 encephalitis. This could lead to an underestimation of ACV penetration, as prior studies have shown a positive correlation between BBB permeability and ACV penetration during encephalitis (16). Additionally, the current study was conducted on a porcine model, and although pigs share significant anatomical and physiological similarities with humans, interspecies differences in ACV tissue disposition may limit the translational applicability of our findings (39). To our knowledge, this is the first study to analyze ACV pharmacokinetics in pigs. Potential physiological differences, such as higher CSF turnover rates and different brain-to-CSF volume ratios in pigs, may result in shorter half-lives of hydrophilic molecules like ACV in pig CSF compared to humans, potentially affecting the extrapolation of our results to clinical settings (19). Unknown to the authors at study conception and conduct, an animal-equivalent dose of 11 mg/kg (adjusted for body surface area) would have been more appropriate than the 10 mg/kg dose used in our study (44). Although we believe that a 10% difference in dosing has a minimal impact on the tissue penetration ratios, it probably has a mild influence on *f*C_max_ and *f*T > IC_50_ values observed.

Furthermore, the bolus intravenous administration of ACV in this study—selected to minimize fluid load and ensure a uniform input rate—differs from the approximately 30 min infusions commonly used in clinical practice and could therefore affect *f*C_max_. This rapid delivery produces a sharper plasma peak and may accelerate early CNS entry compared with slower infusions. Experimental meningitis data indicate that such peaks can transiently elevate CSF concentrations without materially changing total CSF exposure (45). Consequently, the peak penetration ratios reported here might be slightly overestimated, whereas the cumulative CNS AUC is probably preserved; these infusion-rate considerations should be kept in mind when interpreting the results.

The small sample size, particularly during the second and third doses (*n* = 6), introduces variability in ACV concentrations but is in part accommodated by the dense microdialysis sampling. This individual variability has been noted in previous studies involving ACV, both in humans and animal models. Finally, because multiple comparisons were performed throughout these analyses, the relatively high potential for type I errors cannot be excluded. The low number of animals used as a consequence of restrictions due to animal welfare, however, precludes correction for repeated testing.

### Conclusion

This study is the first to investigate the intracerebral distribution of ACV across multiple doses. When administered intravenously at 10 mg/kg every 8 h, ACV achieved adequate target site concentrations (*f*C_max_ > IC_50_ [0.56 µg/mL]) within the uninflamed and uninfected porcine brain ECF and CSF during the second and third doses, successfully meeting the PK/PD target for HSV-1. Future studies should investigate the intracerebral penetration of ACV under inflammatory conditions and assess the efficacy of the current dosing regimen compared to higher individual dosing strategies or alternative infusion strategies.

## SUPPLEMENTAL MATERIAL

Supplemental material aac.01811-24-s0001.docx Description of chemical analytical methods.

ASM does not own the copyrights to Supplemental Material that may be linked to, or accessed through, an article. The authors have granted ASM a non-exclusive, world-wide license to publish the Supplemental Material files. Please contact the corresponding author directly for reuse.
