# Supplementary material for: Distribution of acyclovir in central nervous system compartments: a porcine pharmacokinetic model
Source: Antimicrob Agents Chemother. 2025 Jul 9;69(8):e01811-24. doi: 10.1128/aac.01811-24 (PMC12327012; doi:10.1128/aac.01811-24)
Supplement: Supplemental material — Description of chemical analytical methods. [file aac.01811-24-s0001.docx]

**Supplementary Material**

**Quantification of Acyclovir Concentrations**

Standards and quality controls (QCs) for acyclovir were prepared by diluting stock solutions in 0.1% sodium chloride solution for dialysate samples and in blank porcine plasma for plasma samples. Calibration standards were formulated at concentrations of 1, 5, 10, 25, 50, and 100 µg/mL. QCs were prepared at concentrations of 5, 45, and 80 µg/mL in both matrices.

**Sample Preparation**

**Dialysate Samples**

All dialysate samples, along with QCs and calibration standards, were thawed at room temperature prior to analysis. Blanks (dialysate matrix without acyclovir) and double blanks (pure water) were also prepared. To each well designated for QCs, standards, and blanks, 495 µL of 0.1% sodium chloride solution containing the internal standard (benzylpenicillin) was added, whereas the double blank wells received 495 µL of pure 0.1% sodium chloride solution without the internal standard. Subsequently, 5 µL of each QC and standard solution was added to their respective wells, while 5 µL of pure 0.1% sodium chloride solution was added to the blank and double blank wells. The plate was vortexed for 10 seconds to ensure proper mixing and then centrifuged at 500 × g for 5 minutes to separate any particulates. After centrifugation, 300 µL of the supernatant from each well was carefully transferred to a new deep well plate for LC-MS/MS analysis.

**Plasma Samples**

Plasma samples, QCs, and calibration standards were thawed at room temperature before processing. Blanks (plasma matrix without acyclovir) and double blanks (pure water) were prepared similarly. To each well designated for samples, QCs, standards, and blanks, 495 µL of water containing the internal standard (benzylpenicillin) was added, while the double blank wells received 495 µL of pure water without the internal standard. Then, 5 µL of each QC and standard solution was added to their respective wells; the blank well received 5 µL of pure plasma, and the double blank well received 5 µL of pure water.

For protein precipitation and filtration, 250 µL of each plasma sample was transferred to its respective well in a filter plate. Similarly, 250 µL of each QC, standard, and blank solution was added to the corresponding wells in the filter plate. The filter plate was placed atop a deep well plate and centrifuged at 200 × g for 10 minutes to facilitate filtration; if the plasma did not completely pass through the filter, the centrifugation time was extended accordingly. After filtration, 20 µL of filtrate from each sample well was transferred to a new deep well plate designated for samples, while 200 µL from each QC, standard, and blank well was transferred to their respective positions in the deep well plate. An additional 180 µL of water containing the internal standard was then added to all wells containing samples. The plate was gently shaken for 10 seconds to ensure homogeneity and briefly centrifuged to eliminate any air bubbles prior to LC-MS/MS analysis.

**Liquid Chromatography–Mass Spectrometry Analysis**

Quantitative analysis of acyclovir was performed using high-performance liquid chromatography coupled with tandem mass spectrometry (HPLC-MS/MS). The analytical system consisted of a Shimadzu Exion UHPLC coupled to a SCIEX 4500 QTrap mass spectrometer equipped with an electrospray ionization source. Chromatographic separation was achieved using a Phenomenex Luna Omega C18 column with a particle size of 1.6 µm, dimensions of 50 × 2.1 mm, and a pore size of 100 Å, maintained at a temperature of 65 °C. The mobile phase comprised Milli-Q water with 0.1% formic acid (solvent A) and acetonitrile with 0.1% formic acid (solvent B), delivered at a flow rate of 0.6 mL/min.

The gradient elution program initiated with 10% solvent B at 0.0 minutes. The proportion of solvent B was increased linearly to 80% at 0.90 minutes and then ramped to 95% at 0.95 minutes. It was held at 95% solvent B until 1.35 minutes, after which it was increased to 100% solvent B at 1.35 minutes. The composition was returned to the initial 10% solvent B at 1.36 minutes and maintained until 1.60 minutes, resulting in a total run time of 1.60 minutes per sample. An injection volume of 10 µL was used for all samples. Mass spectrometric detection was performed in positive electrospray ionization mode using multiple reaction monitoring (MRM). The source parameters were optimized with a curtain gas setting of 40 psi, an ion spray voltage of 5,500 V, a temperature of 450 °C, and both ion source gas 1 and ion source gas 2 set at 30 psi. The entrance potential was set to 10 V.

For acyclovir, two ion transitions were monitored. The quantifier ion transition was m/z 226.1 → 152.1, with a declustering potential of 25 V, a collision energy of 18.8 V, and a collision cell exit potential of 10 V. The qualifier ion transition was m/z 226.1 → 135.0, using the same declustering potential of 25 V, a collision energy of 41 V, and a collision cell exit potential of 10 V. Benzylpenicillin, used as the internal standard, was monitored with transitions m/z 335.0 → 160.0 and m/z 335.0 → 176.0, utilizing a declustering potential of 72 V, a collision energy of 18 V, and a collision cell exit potential of 6 V.

**Method Validation**

The analytical method was validated for linearity, precision, accuracy, and sensitivity. Calibration curves demonstrated linearity over the concentration range of 1–100 µg/mL, with correlation coefficients (r²) consistently exceeding 0.99. Intra-day and inter-day precision were evaluated by analyzing QC samples at concentrations of 5, 45, and 80 µg/mL in triplicate over multiple days. The coefficients of variation (CV%) were calculated and found to be less than 5% across all QC levels, indicating high precision and reproducibility.

The lower limit of quantification (LLOQ) for acyclovir was determined to be 0.01 µg/mL, ensuring the method's sensitivity for detecting low concentrations of the analyte.
